# Physical activity moderates the deleterious relationship between cardiovascular disease, or its risk factors, and quality of life: Findings from two population-based cohort studies in Southern Brazil and South Australia

**DOI:** 10.1371/journal.pone.0198769

**Published:** 2018-06-07

**Authors:** Viviane de Menezes Caceres, Nigel Stocks, Robert Adams, Dandara Gabriela Haag, Karen Glazer Peres, Marco Aurélio Peres, David Alejandro González-Chica

**Affiliations:** 1 Discipline of General Practice, Adelaide Medical School, NHMRC Centre of Research Excellence to Reduce Inequality in Heart Disease, The University of Adelaide, Adelaide, South Australia, Australia; 2 Postgraduate Program in Rehabilitation Sciences, Department of Health Sciences, Federal University of Santa Catarina, Araranguá, Santa Catarina, Brazil; 3 The Health Observatory, Adelaide Medical School, The University of Adelaide, Adelaide, Australia; 4 School of Public Health, The University of Adelaide, Adelaide, South Australia, Australia; 5 Australian Research Centre for Population Oral Health (ARCPOH), Adelaide Dental School, The University of Adelaide, Adelaide, South Australia, Australia; University of Zurich, SWITZERLAND

## Abstract

**Background:**

Few studies have investigated the relationship between physical activity (PA) of low intensity and duration with quality of life (QoL) among individuals at risk or with cardiovascular disease (CVD).

**Objectives:**

To investigate whether PA of different intensity and duration moderates the relationship between CVD and its risk factors (obesity, hypertension, diabetes, dyslipidaemia) and QoL in adults.

**Methods:**

Population-based cross-sectional studies using data from the EpiFloripa Cohort Study (Southern Brazil; n = 1,220, 38.8±12.0 years, 48.2% males) and the North West Adelaide Health Study (NWAHS, South Australia; n = 1,661, 43.7±11.1 years, 49.7% males). The physical and psychological domains of QoL were assessed using the WHOQOL-Bref (EpiFloripa) or the SF-36 (NWAHS) questionnaires. The diagnosis of CVD and its risk factors were self-reported. PA was self-reported and quantified by its intensity [“walking” or moderate/vigorous (MVPA)] and duration (none, 1–150, ≥150 min/week). Both studies were analysed separately, and results were adjusted for sociodemographic variables.

**Results:**

Participants at risk or with CVD from both studies showed a lower QoL than ‘healthy’ individuals with a stronger relationship for the physical domain. PA duration showed a direct-trend relationship with QoL, but the associations were stronger for MVPA in both studies. However, when stratified by health status, the magnitude of the association between “walking” duration and a higher physical QoL was greater among those at risk or with CVD compared to ‘healthy’ individuals. Conversely, among Australians with CVD, MVPA was associated with a better physical QoL only when its duration was ≥150 min/week. All associations were stronger in the NWAHS than in the Brazilian study.

**Conclusions:**

“Walking” was more prevalent than MVPA and was consistently associated with a better physical QoL among those at risk or with CVD. These findings should be considered in the design of public health interventions designed to increase PA and improve QoL.

## Introduction

Worldwide, the burden of cardiovascular disease (CVD) and its risk factors have taken centre stage in noncommunicable disease (NCD) policies [1, 2]. CVD accounts for 31% of global deaths. While the majority of these deaths occur in low and middle-income countries, the proportion of deaths due to CVD when compared to other causes is even greater among high-income countries [1, 2]. According to the World Economic Forum, NCDs will cause a global loss of US$ 47 trillion over the following two decades, with CVD being the most important contributor [3]. Moreover, although the prevalence of some NCDs has recently declined in many high- and some middle-income countries, CVD mortality has plateaued, especially among the high-income countries [4].

CVD also has a profound impact on physical and mental health, self-esteem, and the daily lives of affected individuals [5–7]. The Global Burden of Disease estimates that between 1990 and 2010 the disability-adjusted life-year (DALYs) of CVDs increased by 22.6%, becoming the leading cause of DALYs worldwide [8, 9]. In this scenario, appropriate long-term management of CVD is fundamental for reducing acute events, further complications, and health expenditure, as well for improving life expectancy and quality of life (QoL) [10].

Among the non-pharmacological strategies for the prevention and management of patients at risk of—or with—CVD, physical activity (PA) has been shown to be beneficial for improving physical health and physiological parameters, as well as reducing complications and mortality [11–15]. The World Health Organization recommends that adults should perform at least 150 minutes/week of moderate PA or 75 minutes/week of vigorous PA (MVPA) or an equivalent combination of both as this is associated with a reduced risk of developing diverse NCDs and premature mortality in the general population [16]. Nonetheless, some studies suggest that these recommendations may be difficult to achieve among individuals with functional limitations, such as those affected by CVD, and lower levels of PA may still be beneficial for reducing the risk of coronary heart events and encouraging individuals to integrate PA as part of their daily lifestyle [12, 17]. Furthermore, PA seems to improve other health outcomes among patients with CVD, including different domains of QoL [13, 18].

QoL is a subjective outcome that assesses individuals’ perceptions of the impact of diverse conditions on their daily living, including their social and cultural context [19]. This patient-centred outcome has become increasingly important in the study of CVD due to its close relationship with physical functioning, adherence to health management, hospitalisations, and mortality [20]. PA has been consistently associated with a better QoL in the general adult population [21]. However, the impact of PA on QoL among individuals at risk or with CVD has not been sufficiently evaluated in population-based studies. Furthermore, most research in this field has been conducted in hospital/clinical settings from high-income countries, especially among those located in the Northern hemisphere [18, 22–24].

Therefore, investigating the association between PA of different intensity and duration and the QoL among individuals at risk of, or with, CVD using population-based samples is crucial to be able to give more accurate advice about the volume and intensity of PA that can improve health in different socioeconomic contexts. In this sense, Brazil and Australia are two countries in the Southern hemisphere with a similar profile in terms of CVD mortality (33% and 35% of all deaths, respectively), risk factors (prevalence of diabetes mellitus being 8% in both countries, overweight being 54% and 66% respectively, and hypertension being 23% and 15%, respectively) as well as lifestyle characteristics (prevalence of smoking being 17% and 16% respectively, and insufficient PA being 84% in both countries) [25, 26]. There is also universal access to a comprehensive range of health services in both countries, including community and emergency health services [27–30]. Nonetheless, Brazil and Australia, as middle-income and high-income countries, are very different according to other indicators such as life-expectancy (73 in Brazil vs 82 years in Australia) and the human development index (0.754 in Brazil vs 0.939 in Australia) [31].

Therefore, in this study we used data from two population-based cohort studies from Southern Brazil and South Australia to explore and compare the association between CVD and CVD risk factors on QoL, as well as assess whether PA of different intensity and duration moderates that relationship.

## Material and methods

### Study design and setting

This is a cross-sectional study using data from the EpiFloripa Adult Cohort Study (Florianópolis, Southern Brazil) and the North West Adelaide Health Study (NWAHS, Adelaide, South Australia). The Brazilian cohort study was conducted between 2009–2014 and included 1,720 participants aged 20–59 years at the baseline. The Australian cohort study was conducted between 2000–2010 and involved 4,056 individuals aged 18–95 years at the baseline. Ethics approval was obtained from the Human Research Ethics Committees at the Federal University of Santa Catarina (Brazil) and The University of Adelaide (Australia). All participants signed their respective consent forms.

### Data collection/participants

A random sampling of the population was used to select participants in both studies and details have been published elsewhere (S1 Fig) [32, 33]. In summary, the target population in the baseline of the EpiFloripa study (2009–2010) included individuals living in the urban area of Florianópolis (97% of the inhabitants), a state capital in Southern Brazil. Firstly, ten census sectors were systematically selected in each decile of household income (63/420 sectors), and then 1,134/16,755 households in these sectors were systematically selected. Excluding individuals with severe mental or physical disabilities, all adults aged 20–59 years living in these households were eligible for the study. Considering a mean of 1.78 per dwelling, the sampling process allowed the identification of 2,016 adults. All participants evaluated in the baseline (N = 1,720) were traced in 2012–2013 (second wave) and 71% were interviewed (S1 Fig). In both waves, participants were home interviewed and data were collected using face-to-face questionnaires with direct measurement of clinical and anthropometric variables.

All households in Northern and Western Adelaide connected to a landline telephone were considered eligible in the baseline of the NWAHS (2000–2003; 97.9% of households in these areas). The target population represented half of the 1.1 million inhabitants in the metropolitan area of Adelaide and one-third of the population in South Australia. One adult aged 18+ years living in the randomly selected households was arbitrarily selected (excluding those unable to communicate in English or with severe illness). Data were collected using a combination of phone interviews (Computer Assisted Telephone Interviews), self-administered questionnaires posted to the address of the participants, and clinical assessments at two Central Northern Adelaide Health Service health centres. A total of 4,056 individuals of the 8,213 eligible individuals completed the interviews and attended the clinics. The second and third waves of the study were performed in 2006–2007 (n = 3,564) and 2008–2010 (n = 2,871) respectively. Analyses of the NWAHS for this study were restricted to those interviewed in 2008–09 and aged <65 years (n = 1,661) to get a comparable sample with the Brazilian study (aged 23–63 years in 2012).

### Outcome: Quality of life

In the EpiFloripa study, QoL was measured in 2012–2013 (second wave) using the Portuguese version of the World Health Organization Quality Instrument for Quality of Life Assessment (WHOQOL-Bref). This 26-item instrument generates four 0-100-scale domains (physical, psychological, environmental, and social relationships) [34].

For the NWAHS, the physical and mental component scores of QoL were assessed in 2008–2010 (third wave) using the 36-Item Short Form Survey (SF-36) [35, 36]. The questionnaire consists of 36 questions, which generate eight sub-domains (physical functioning, role-physical, bodily pain, general health, vitality, social functioning, role-emotional, and mental health). These sub-domains were combined to generate the physical and mental component scores using recommended algorithms [35].

WHOQOL-Bref and SF-36 were developed to measure different constructs (QoL or Health-related QoL, respectively), include different domains, and are expressed in different scales (WHOQOL-Bref_Mean_ = 70 points; SF-36_Mean_ = 50 points) [19, 37]. Nonetheless, both instruments have consistently identified the adverse relationship of CVD and their risk factors on QoL, as well as the benefits of PA levels on that outcome. Therefore, to obtain comparable results, only the physical and psychological domains of the WHOQOL-Bref were analysed. Additionally, for this study, we will use the terms “physical” or “psychological” domains of QoL independent of the instrument used to measure these outcomes in each study.

### Clinical conditions: Cardiovascular disease and risk factors

In both studies, information collected in different waves were used to identify those individuals at risk of or with CVD (baseline in the EpiFloripa study and at baseline and third wave of the NWAHS) (S1 Fig). Individuals at risk were defined as those with hypertension, dyslipidaemia, diabetes mellitus, obesity, and/or abdominal obesity, but not CVD [25, 26]. Obesity was defined as a body mass index (BMI) ≥30 kg/m^2^ based on measured weight and height (EpiFloripa, 2012–2013; NWAHS, 2008–2010). Abdominal obesity was defined as being in the upper quartile of waist circumference separately for each sex (EpiFloripa = men >99.0 cm and women >89.6 cm; NWAHS >108.3 cm and >99.1 cm, respectively). The recommended WHO cut-off points for waist circumference were not used because of the lack of consensus for different ethnic groups; for example, overestimating the prevalence of abdominal obesity among Latin-American women by more than 30% [38].

The other risk factors were identified based on self-reported medical diagnoses of these conditions (“*Has a medical doctor ever told that you have*…?”) and combined with the use of medication for these conditions. Similarly, participants were questioned about previous diagnoses of CVD (i.e. myocardial infarction, angina, heart failure, atrial fibrillation/arrhythmia, and/or stroke) using data from the baseline in the EpiFloripa study and third wave in the NWAHS. Based on all this information, individuals were classified as: 1) negative for all these conditions (None), 2) at risk of CVD (but without CVD), or 3) with CVD (with or without a risk factor).

### Moderator: Physical activity levels

PA was measured in the baseline of the EpiFloripa study using questions from the Surveillance of Risk and Protective Factors for Non-Transmissible Chronic Diseases by Telephone Interviews (VIGITEL) [39]. In the NWAHS, PA was assessed in the third wave using items from the Active Australia Questionnaire (AAQ) [40, 41]. Both instruments have shown good reliability and validity when compared to gold standard methods in the assessment of PA levels (moderate correlations, sensibility 50–70%, specificity of at least 80%). In both studies, PA was self-reported and based on different questions regarding the weekly volume (number of days and total time) and intensity (light, moderate, vigorous) of leisure and/or transportation activities performed regularly (over the past three months in Brazil and over the last week in Australia). For analytical purposes, “walking” for recreation, exercise, or transportation was analysed separatedly, as it could include moderate but also low intensity PA (activities with a a metabolic equivalent (1 MET = 1 kcal/kg/hour) < 3.0) [42]. “Moderate intensity” PA was defined as exercise causing a moderate increase in heart rate or breathing (equivalent to 3.0–5.0 MET), such as running, lawn bowls, golf, swimming, dancing, or hydrogymnastics [39–42]. “Vigorous intensity” was defined as exercise causing a large increase in a person’s heart rate or breathing (equivalent to >5.0 MET) such as jogging, cycling, soccer, tennis, basketball, and keep-fit exercises [39–42]. Both moderate activities and vigorous activities were combined as MVPA. Following the WHO PA recommendations for adults [16], the total volume of “walking” or MVPA (with “vigorous” multiplied by two to account for its greater intensity) each was classified as 1) none (no PA reported), 2) <150 min/week, or 3) ≥150 min/week.

### Covariates

Sociodemographic variables were included as possible confounding factors of the association between clinical conditions and QoL; these were selected based on findings from the literature [14, 43–48]. Most of these variables were collected in the baseline of the EpiFloripa study or third wave of the NWAHS (S1 Fig), and they included sex (male or female), age (continuous variable, but also analyzed including a quadratic term due to nonlinear relationship with QoL), marital status [married (includes living with a partner), single, or divorced/widowed], educational level [up to secondary school, trade/certificate, or bachelor and higher (equivalent to up to 11 years, 12–14 years, or 15+ years, respectively)], and family income. Family income was collected as a continuous variable and classified into quartiles in the Brazilian study. In the NWAHS, it was collected in eight categories, which were re-grouped into four [up to $20,000 (lowest), $20,001–$40,000 (low-middle), $40,001–$60,000 (middle-high), or more than $60,000 (highest)] to obtain a more balanced distribution between categories.

### Data analysis

All analyses were performed separately for both studies, considering sampling weights (probability of selection in the baseline, probability of location in the follow-up, and re-weighted to the population distribution) and the survey design in each study [32, 33].

For descriptive analyses, categorical variables were presented as percentages (%), while numerical variables were described as means with their standard deviation (SD) or median with interquartile range (p25-p75), depending on their distribution. Bivariate analyses between sociodemographic variables, PA, and QoL (a continuous outcome) were performed with t-tests or ANOVAs (the heterogeneity or trend depending on the nature of the independent variables).

To test the association between clinical conditions (none, at risk of CVD, or with CVD) and the physical and psychological domains of QoL, the outcomes of each study were standardised (Mean = 0, SD = 1) using the sample distribution to provide comparable results. Linear regressions were then used to evaluate the crude and adjusted associations between these variables. Regression coefficients (β) and the respective 95% confidence intervals (95%CI) obtained from these analyses were interpreted as the difference of QoL in SD between those at risk or with CVD compared to the reference category (“none” of them). Sociodemographic variables were included in the models as possible confounds [45, 46, 49], independent of their level of statistical significance in the association with the outcomes. The variance inflation factor (VIF) was investigated as an indicator of multicollinearity between the explanatory variables.

The moderating role of PA was tested by the inclusion of multiplicative terms between “walking” or MVPA time with the clinical health status. A p-value <0.10 for the multiplicative term was considered as indicative of heterogeneity of the associations [50]. Predicted adjusted means of QoL in each category of the PA level were then estimated and presented graphically with their 95%CI, stratified by the respective clinical health status.

Coefficients of determination (R^2^) were used to assess the variance of QoL explained by the models. All analyses were used the STATA software, version 13.0 (StataCorp, Texas, USA).

## Results

Of the 1,720 adults included in the baseline of the EpiFloripa study, and the 4,060 participants in the NWAHS, 71.0% and 70.7% were located in the last wave of each study (S1 Fig). Located participants were comparable to the baseline sample regarding sex, age, marital status, educational level, and nutritional status (S1 Table). Thus, the Brazilian study included 1,222 adults (38.8 ± 12.0 years, 48.2% males) and the Australian study included 1,661 individuals aged <65 years (43.7 ± 11.1 years, 49.7% males). Table 1 summarizes the sociodemographic and clinical characteristics of the participants in each study.

**Table 1:** Prevalence of sociodemographic and clinical variables among adults in the EpiFloripa study (Southern Brazil, 2012–2013) and North West Adelaide Health Study (South Australia, 2008–2010).

Variables	EpiFloripa (n = 1,222)		NWAHS (n = 1,661)	
	%	95% CI	%	95% CI
**Sex: Males**	48.2	45.1;51.4	49.7	46.3;53.1
**Age**				
23 to 29 years	30.3	25.9;35.2	12.6	9.6;16.5
30 to 39 years	24.9	21.8;28.3	26.7	23.3;30.4
40 to 49 years	21.6	18.9;24.5	27.2	24.6;29.9
50 to 64 years	23.2	19.8;27.0	33.5	30.9;36.3
**Marital status**				
Married	57.1	53.1;60.9	73.5	70.0;76.7
Single	34.6	30.6;38.9	16.9	13.8;20.5
Divorced/Widowed	8.3	6.6;10.4	9.6	8.2;11.3
**Attained educational level**				
Up to secondary	55.6	48.1;62.9	40.8	37.6;44.2
Certificate/Diploma	14.0	11.2;17.2	35.1	31.8;38.5
Bachelor or higher	30.4	24.4;37.2	24.1	21.0;27.5
**Family income**#				
Lowest	28.5	23.7;34.0	18.5	16.1;21.1
Low-middle	21.2	17.5;25.5	17.7	15.3;20.4
Middle-high	26.0	22.6;29.8	20.9	18.1;24.1
Highest	24.2	18.7;30.8	42.9	39.3;46.5
**Walking**				
None	45.3	41.1;49.6	26.0	23.0;29.2
1–149 min/week	39.4	35.2;43.7	48.8	45.3;52.3
≥150 min/week	15.3	12.9;18.1	25.3	22.4;28.3
**Moderate/vigorous PA**				
None	65.9	61.0;70.4	59.9	56.4;63.4
1–149 min/week	16.3	13.8;19.1	25.7	22.6;29.0
≥150 min/week	17.9	14.0;23.4	14.4	12.0;17.2
**Risk factors (% Yes)**				
Hypertension^a^	14.7	12.3;17.5	13.8	12.2;15.7
Dyslipidemia^a^	4.5	3.2;6.2	9.5	8.2;11.0
Diabetes mellitus^a^	3.6	2.7;4.9	4.6	3.7;5.7
Obesity^b^	18.7	15.7;22.1	34.0	30.9;37.3
Abdominal obesity^c^	22.6	19.5;26.0	21.7	19.1;24.4
**Clinical health status**				
None	63.4	58.9;67.6	59.1	55.8;62.4
At risk of CVD*	29.9	26.4;33.8	36.1	33.0;39.4
With CVD**	6.7	5.3;8.4	4.8	3.8;6.1

^**#**^ Brazil: continuous variable presented in quartile. Australia: family income was collected in eight categories which were re-grouped to obtain a better balance between categories (up to $20,000 (Lowest), $20,001–$40,000 (Low-middle), $40,001–$60,000 (Middle-high), or more than $60,000 (Highest)).

^a^—Based on medical diagnosis and/or medical treatment for that specific condition

^b^—Body mass index (BMI) ≥30 kg/ m^2^, based on measured weight and height in Brazil and self-related in Australia

^c^—Upper quartile of waist circumference (Brazil: men >99.0 cm and women >89.6 cm; Australia: men >108.4 cm and women >99.2 cm)

*Individuals with hypertension and/or dyslipidaemia and/or diabetes and/or obesity and/or abdominally obese, but without cardiovascular diseases

** Including myocardial infarction, angina, heart failure, atrial fibrillation/arrhythmia, and/or stroke (with or without cardiovascular diseases risk factor)

The proportion of participants with at least 150 min/week of “walking” was higher in the NWAHS, while MVPA of the same duration was lower in the same study. The prevalence of hypertension and diabetes mellitus was similar in both studies, but the frequency of dyslipidaemia and obesity in the NWAHS was almost twice as high as in the EpiFloripa study. Conversely, the prevalence of CVD was higher in the Brazilian study. Nonetheless, the frequency of individuals without any risk factor or CVD (“none”) was similar in both studies.

**Fig 1:**
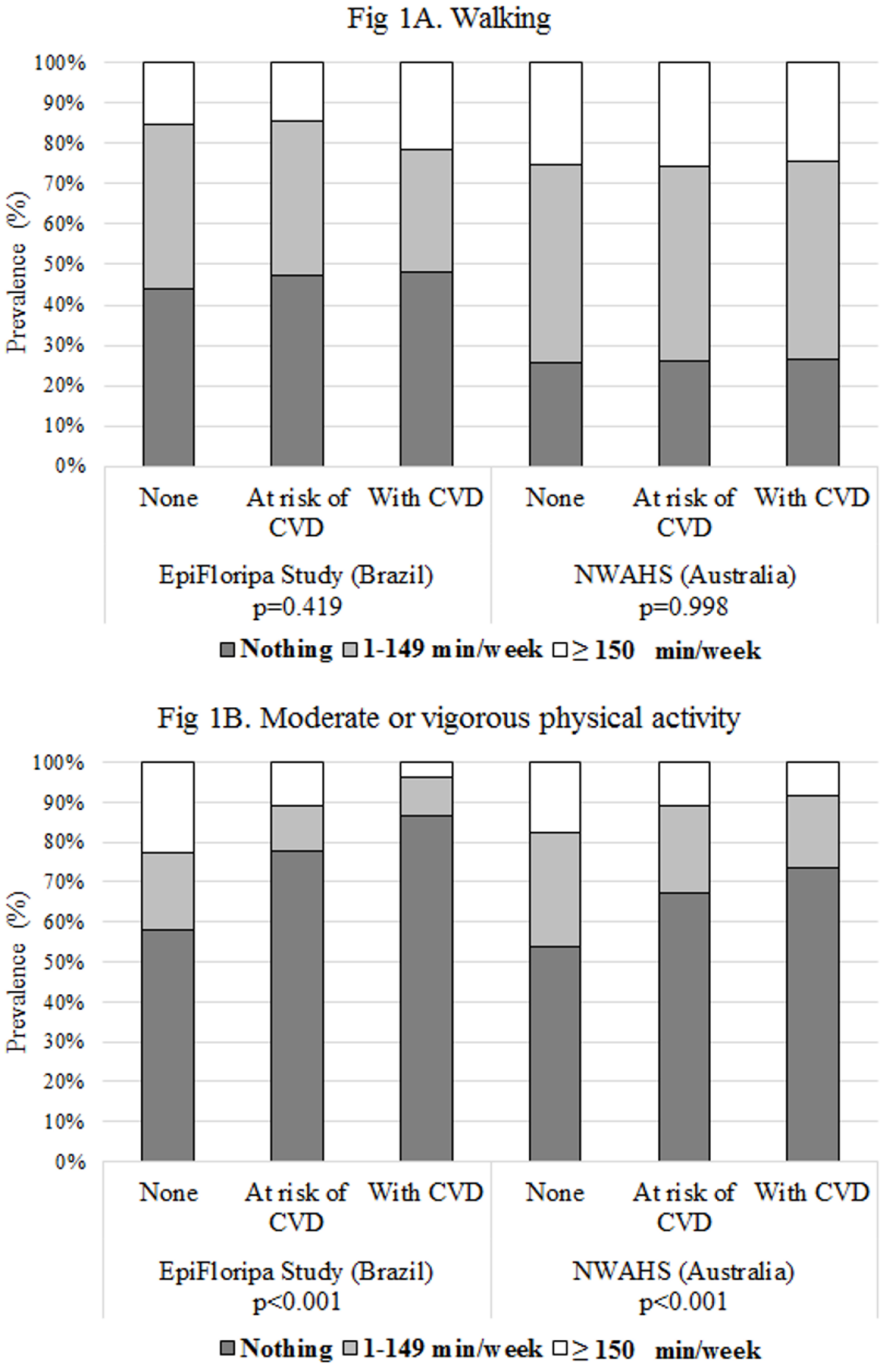
Prevalence of PA according to the clinical health status among adults in the EpiFloripa study (Southern Brazil) and North West Adelaide Health Study (NWAHS, South Australia). (A) “Walking”. (B) Moderate/vigorous PA. At risk of CVD: Individuals with hypertension and/or dyslipidemia and/or diabetes and/or obesity and/or abdominal obesity, but without cardiovascular diseases. With CVD: Including myocardial infarction, angina, heart failure, atrial fibrillation/arrhythmia, and/or stroke (with or without risk factor).

Fig 1 shows the prevalence of PA according to the clinical health status. In both studies, the levels of “walking” were similar independent of whether the participants had a clinical history of CVD or their risk factors (A). On the other hand, the percentage of individuals performing any level of MVPA reduced progressively depending on whether they were at risk or affected by a CVD (B). Nonetheless, this reduction was more evident in the EpiFloripa Study as the frequency of individuals with CVD practicing some level of MVPA (1–149 min/week = 9.9% and ≥150 min/week = 3.7%) was half the prevalence observed in the Australian study (8.3% and 18.2%, respectively).

Regarding the QoL domains, even though a different instrument was used in each study, the physical domain was slightly higher than the psychological domain, both in the Brazilian (73.8 ± 6.2 and 71.6 ± 4.6, respectively) and in the Australian study (49.7 ± 8.2 and 47.6± 8.8, respectively).

Table 2 shows the mean scores of QoL according to the sociodemographic and clinical variables. In general, the physical and psychological domains in both studies were higher in males, younger individuals (there was no association with age for the psychological domain in Australia), with a higher educational level, or higher family income. “Walking” of any duration was associated with a better physical and psychological QoL in the Australian sample, but no association with any domain was observed in the Brazilian study. However, in general, the highest physical and psychological QoL scores were observed among individuals in both studies practicing ≥150 min/week of MVPA.

**Table 2:** Physical and psychological domains of quality of life according to sociodemographic and clinical characteristics in the EpiFloripa study (Southern Brazil, 2012–2013) and North West Adelaide Health Study (South Australia, 2008–2010).

	Physical domain				Psychological domain			
	Epifloripa		NWAHS		Epifloripa		NWAHS	
	Mean	95% CI	Mean	95% CI	Mean	95% CI	Mean	95% CI
**Sex**		p<0.001*		p = 0.005*		p = 0.001*		p = 0.001*
Male	76.6	75.3;77.9	50.5	49.8;51.2	73.0	71.9;74.1	48.6	47.8; 49.4
Female	71.3	69.6;72.9	49.0	48.2;49.8	70.2	68.9;71.5	46.6	45.7; 47.5
**Age group**		p<0.001**		p<0.001**		p = 0.004**		p = 0.700
20 to 29 years	77.3	75.6;79.0	51.9	50.0;53.8	73.3	71.6;74.9	48.2	45.6;50.8
30 to 39 years	74.0	71.9;76.0	50.3	49.1;51.6	71.5	69.3;73.7	47.1	45.6;48.6
40 to 49 years	73.4	71.5;75.4	50.3	49.5;51.2	71.9	70.3;73.4	48.0	47.0;49.1
50 to 64 years	69.5	66.8;72.2	47.9	47.2;48.6	69.1	67.5;70.7	47.4	46.8;48.1
**Marital status**		p = 0.020***		p = 0.010***		p = 0.150		p<0.001***
Married	73.1	71.6;74.6	50.0	49.4;50.6	71.1	70.0;72.3	48.2	47.6;48.8
Single	75.6	73.8;77.4	49.9	48.0;51.7	72.6	70.8;74.5	46.9	44.8;49.1
Divorced or widowed	71.5	67.8;75.3	47.2	45.5;48.9	70.0	67.0;73.0	44.0	42.0;46.0
**Educational level**		p<0.001**		p<0.001**		p<0.001**		p = 0.003**
Up to secondary	71.8	70.2;73.5	48.5	47.7;49.3	69.0	67.7;70.3	46.6	45.7;47.6
Certificate or Diploma	75.6	73.4;77.8	49.5	48.5;50.4	74.3	71.6;77.0	47.8	46.6;48.9
Bachelor or higher	76.5	75.1;78.0	52.1	51.0;53.1	75.0	73.9;76.1	49.0	47.8;50.1
**Family income**^a^		p<0.001**		p<0.001**		p<0.001**		p<0.001**
Lowest	69.7	67.7;71.1	44.8	43.4;46.2	67.3	65.9;68.8	43.1	41.5;44.6
Low-middle	73.4	70.9;75.9	49.4	48.0;50.8	71.9	70.0;73.8	46.9	45.2;48.6
Middle-high	74.4	72.5;76.4	50.3	49.0;51.7	72.5	70.5;74.4	47.1	45.6;48.7
Highest	77.8	76.1;79.6	52.0	51.4;52.6	75.2	73.8;76.6	50.3	49.6;50.9
**Walking**		p = 0.060		p<0.001**		p = 0.848		p = 0.006
None	72.8	70.7;74.8	48.3	47.2;49.4	71.8	70.4;73.2	45.9	44.5;47.3
1–149 min/week	74.1	72.6;75.6	50.0	49.2;50.9	71.2	69.9;72.6	48.1	47.2;49.0
≥150 min/week	76.4	73.9;78.8	50.7	49.8;51.6	71.7	69.2;74.2	48.4	47.3;49.5
**Moderate/vigorous PA**		p<0.001**		p<0.001**		p<0.001**		p<0.001**
None	72.5	71.0;74.0	48.3	47.5;49.0	70.6	69.5;71.6	46.7	45.9;47.5
1–149 min/week	76.5	74.2;78.8	51.2	50.1;52.2	72.5	70.1;75.0	47.9	46.5;49.3
≥150 min/week	76.4	74.1;78.6	53.3	52.3;54.2	74.3	72.8;75.9	50.9	49.6;52.2
**Clinical health status**		p<0.001**		p<0.001**		p = 0.002**		p<0.001**
None	76.9	75.8;78.1	51.3	50.6;52.0	73.3	72.2;74.4	48.8	47.9;49.6
At risk of CVD^b^	69.3	67.4;71.2	47.7	46.8;48.5	68.5	66.9;70.0	46.0	45.0;47.0
With CVD^c^	65.2	59.6;70.8	45.4	43.1;47.7	68.9	65.7;72.1	45.2	42.5;47.8

P-value <0.05 according to a * t-test, ** an ANOVA test for trend, or an ***ANOVA test for heterogeneity

^a^—Brazil: continuous variable presented in quartile. Australia: family income was collected in eight categories, which were re-grouped aiming to obtain a better balance between categories (up to $20,000 (Lowest), $20,001–$40,000 (Low-middle), $40,001–$60,000 (Middle-high), or more than $60,000 (Highest)).

^b^–Individuals with hypertension and/or dyslipidaemia and/or diabetes and/or obesity and/or abdominally obese, but without cardiovascular diseases

^c^–Including myocardial infarction, angina, heart failure, atrial fibrillation/arrhythmia, and/or stroke (with or without cardiovascular diseases risk factor)

Table 3 shows the crude and adjusted associations between clinical health status and QoL (standardised scores, as detailed in the Methods section). Results for the physical domain were consistent in both studies in terms of magnitude and direction of the associations. After adjustment for confounding variables, individuals at risk of CVD showed a physical score 0.3 SD lower than the reference category (“none”), while among those with CVD the score was 0.5 SD lower. The psychological domain in both studies was also lower among individuals at risk or with CVD, either in crude or adjusted analyses. However, the magnitude of the association with CVD was stronger in the Australian study (-0.43 SD; 95%CI -0.75;-0.11) compared to the EpiFloripa study (-0.17 SD; 5%CI -0.40;-0.07), with no inverse-trend association between the clinical health status and the psychological domain in the Brazilian study. No evidence of multicollinearity between the explanatory variables was identified, as the mean VIF did not exceed 1.25 in any model.

**Table 3:** Crude and adjusted analyses^1^ of the association between clinical health status and quality of life (physical and psychological domains) in the EpiFloripa study (Southern Brazil, 2012–2013) and North West Adelaide Health Study (South Australia, 2008–2010).

		Physical domain				Psychological domain			
		Crude		Adjusted^a^		Crude		Adjusted^a^	
		β (95% CI)		β (95% CI)		β (95% CI)		β (95% CI)	
EpiFloripa	None	Ref		Ref		Ref		Ref	
	At risk of CVD^b^	-0.46	(-0.58;-0.34)	-0.37	(-0.50;-0.24)	-0.34	(-0.48;-0.21)	-0.27	(-0.41;-0.13)
	With CVD^c^	-0.71	(-1.04;-0.38)	-0.54	(-0.87;-0.22)	-0.31	(-0.54;-0.08)	-0.17	(-0.40;-0.07)
	p-value*	p<0.001		p<0.001		p<0.001		p<0.001	
NWAHS	None	Ref		Ref		Ref		Ref	
	At risk of CVD^b^	-0.42	(-0.55;-0.30)	-0.31	(-0.43;-0.19)	-0.31	(-0.46;-0.17)	-0.25	(-0.39;-0.11)
	With CVD^c^	-0.69	(-0.97;-0.41)	-0.52	(-0.80;-0.24)	-0.42	(-0.74;-0.09)	-0.43	(-0.75;-0.11)
	p-value*	p<0.001		p<0.001		p = 0.006		p<0.001	

^1^ –Regression coefficients (β) and 95%CI are presented as standardised units, and should be interpreted as the difference of QoL in standard deviations (SD) between those at risk or with CVD compared to the reference category (“none” of them). Physical domain—EpiFloripa_WHOQoL_ SD = 6.2 and NWAHS_SF-36_ SD = 8.2. Psychological domain—EpiFloripa_WHOQoL_ SD = 4.6 and NWAHS_SF-36_ = 8.8.

^a^–Adjusted for variables sex, age, marital status, attained education level, and family income.

^b^–Individuals with hypertension and/or dyslipidemia and/or diabetes and/or obesity and/or abdominally obese, but without cardiovascular diseases

^c^–Including myocardial infarction, angina, heart failure, atrial fibrillation/arrhythmia, and/or stroke (with or without cardiovascular diseases risk factor)

* P-value for trend

**Fig 2:**
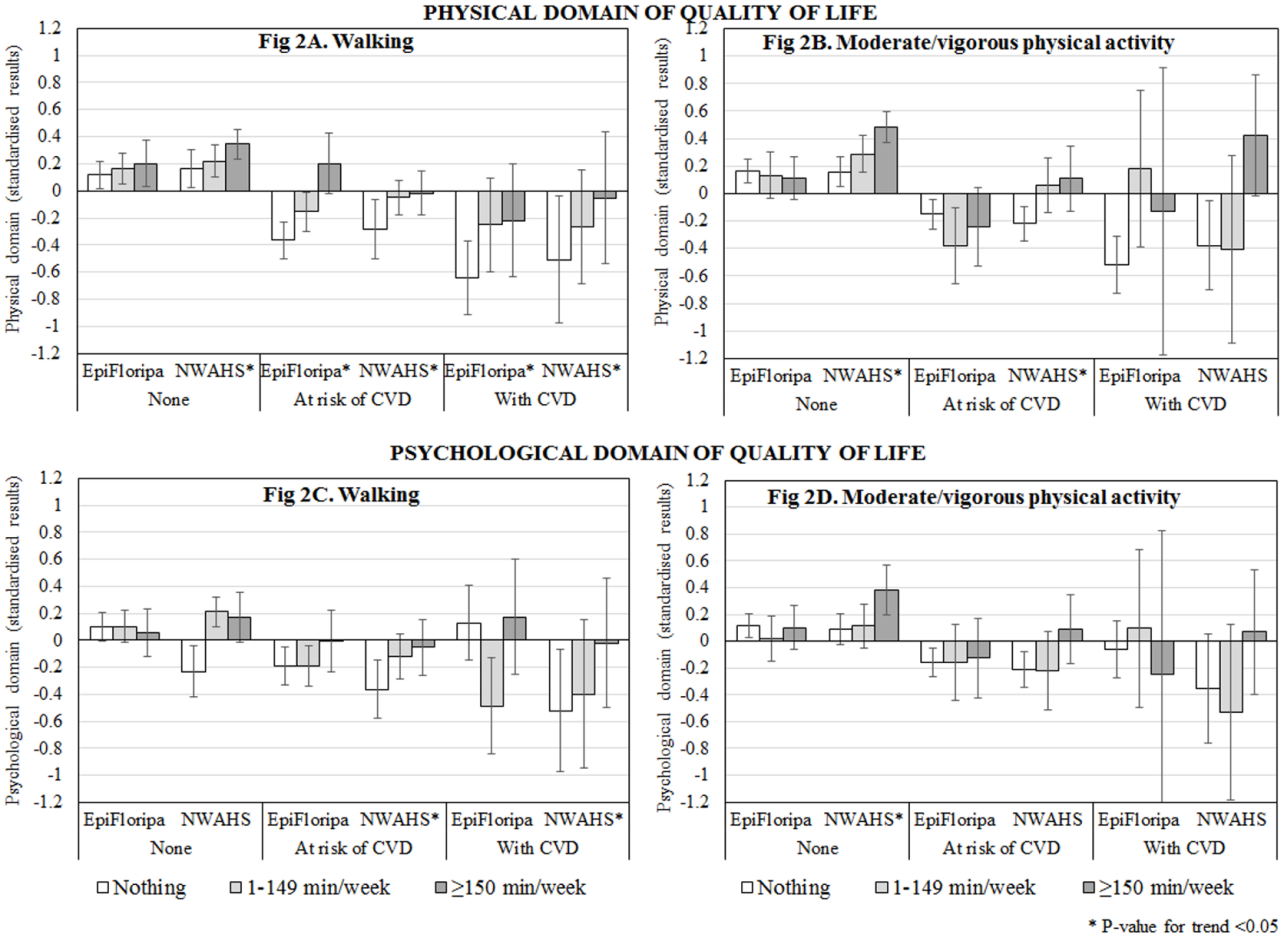
Predicted adjusted means of the physical and psychological domains of quality of life (standardised results) according to the clinical health status and physical activity level in the EpiFloripa study (Southern Brazil, 2012–2013) and North West Adelaide Health Study (South Australia, 2008–2010). Results are adjusted for sex, age, marital status, attained education level, family income, and mutual adjustment between “walking” and MVPA. Individuals at risk include those with hypertension, dyslipidaemia, diabetes, general obesity and/or abdominal obesity, but without CVD. CVD include myocardial infarction, angina, heart failure, atrial fibrillation/arrhythmia, and/or stroke (with or without a risk factor). Quality of life scores were standardised so that the variables have a mean = 0 and SD = 1 (original variables Physical domain EpiFloripa_WHOQoL_ = 73.8±6.2 and NWAHS_SF-36_ = 49.7±8.2; Psychological domain EpiFloripa_WHOQoL_ = 71.6±4.6 and NWAHS_SF-36_ = 47.6±8.8). Vertical lines at the top of the columns represent the 95%CI.

The moderating role of the PA levels on the association between the clinical health status and QoL is shown in Fig 2. In general, “walking” time was directly associated with the physical score of QoL in both studies, independent of clinical health status (A). However, the difference between individuals practicing ≥150 min/week and those who were inactive was higher among participants at risk or with a CVD (differences between 0.3 and 0.6 SD, depending on the study) compared to individuals without these conditions (differences between 0.1 and 0.2 SD). Furthermore, individuals at risk or with CVD practicing some level of “walking” tended to show a mean closer to zero (i.e. equivalent to the study mean). The moderating role of MVPA on physical QoL was less evident (B). In the Brazilian sample, MVPA was not associated with a better physical QoL among individuals who were healthy, at risk, or with CVD. In the Australian study, MVPA showed a direct trend association with physical QoL among healthy individuals; it was also beneficial for those at risk of CVD (similar effect for those practicing 1–149 or ≥150 min/week). However, among individuals with CVD, it was associated with better physical QoL only when its duration was ≥150 min/week.

Regarding the relationship with psychological QoL, in the Brazilian study (C) “walking” time was not associated with this outcome among healthy participants or those at risk of CVD; a lower score was observed among individuals with CVD practicing 1–149 min/week. In the Australian study, individuals without any level of “walking” showed the lowest psychological QoL score, especially if affected by CVD, and a direct trend association was observed between “walking” time and psychological QoL among those at risk or with CVD. MVPA was associated with psychological QoL only in the Australian study (D), showing a direct trend association between the two variables among healthy individuals. MVPA was also beneficial for those at risk of CVD practicing ≥150 min/week, but no association was found among individuals with CVD.

Tests of heterogeneity for the moderating role of PA were <0.05 in all cases, except for the association between MVPA and the psychological domain in the EpiFloripa study.

Compared to the models including just sociodemographic variables and clinical health status, the inclusion of the PA variables in the regression models increased the variance of the physical domain of QoL by approximately 20% (adjusted R^2^ changed from 14.3% to 17.2% in the EpiFloripa study and from 16.9% to 19.9% in the NWAHS). For the psychological domain, the change in the adjusted R^2^ due to the inclusion of the PA variables was lower in the Brazilian (R^2^ increased from 10.4% to 11.9%) than in the Australian study (13.8% and 16.3%, respectively).

## Discussion

This paper investigated the association between CVD and their risk factors with QoL in two population-based cohort studies from Southern Brazil and South Australia. It also explored if PA of different intensity and duration moderated that relationship. Four main findings can be highlighted from our results. Firstly, individuals at risk or with CVD from both studies showed a lower QoL than those without these conditions, and this relationship was stronger for the physical domain. Secondly, although the association with the physical and psychological domains of QoL were more evident for MVPA than “walking” time, individuals at risk or with CVD were more likely to practice “walking” than other MVPA, especially in South Australia. Thirdly, the direct trend association between “walking” and a higher physical QoL was stronger among those at risk or with CVD than among those without these conditions in either study. MVPA among individuals with CVD was associated with a better physical QoL only when its duration was ≥150 min/week, and this relationship was observed only in the Australian study. Finally, the moderating role of PA in the relationship between CVD and the psychological domain was more evident in South Australia. Reduced “walking” time was associated with a lower psychological score, and this adverse relationship was greater among those with CVD, while the direct trend relationship between MVPA and psychological QoL was evident only among ‘healthy’ individuals.

Our findings of lower physical scores compared to psychological scores for QoL among individuals at risk or with CVD has been previously reported in the literature [5, 14, 45, 48]. The negative impact of CVD and CVD risk factors on physical QoL can be explained because of the symptoms (i.e. dyspnea, chest pain) and physical functioning limitations (i.e. mobility problems, difficulties to perform daily activities) related to these conditions [6, 7, 51–53]. Nonetheless, depression and anxiety are also common among these patients, and psychological distress (i.e. fear of another heart attack, disruption of daily activities, frustration) and health management issues (overwhelming treatment plans, poor coping after onset of disease) may contribute to a lower psychological QoL [6, 7, 51, 52]. Moreover, although we identified in both studies that the effect of having CVD was stronger than being affected only by their risk factors, both conditions seem to have the same impact on QoL after the age 65 years [54].

Due to the stronger impact on physical health, PA has been recommended for the management and prevention of CVD as it is beneficial for musculoskeletal fitness, reducing adiposity, enhancing physiological parameters, improving symptoms, and reducing morbimortality [11–13, 17, 18, 22, 53]. Furthermore, consistent with our results, PA is also beneficial for improving psychological QoL and reducing adverse psychological stress parameters (depression, anxiety, and hostility) [14, 21, 22, 24, 53].

Although different guidelines recommend exercising at least 150 minutes/week of MVPA for individuals at risk or with CVD [12, 17], in both studies investigated in this study, individuals affected by these conditions were more likely to undertake “walking” than other MVPA. Furthermore, according to our findings, “walking” was more important than other MVPA in determining physical QoL among individuals at risk or with CVD, both in the Australian and Brazilian samples. Although this could be a consequence of a higher frequency of individuals at risk or with CVD “walking” than MVPA, diverse studies have reported that patients benefit from PA programs even when they only include activities of lower intensity and/or duration [13, 17, 22, 23, 43]. In fact, in our study, a dose-response relationship between MVPA and QoL (both physical or psychological domains) was observed only among healthy individuals in the Australian study, while among those with CVD some benefit was observed only when practiced for ≥150 min/week.

The less evident pattern for the associations between PA and QoL in the Brazilian study could be partially explained by the problems experienced by low-and-middle income countries in implementing existing PA national policies [i.e. insufficient workforce, lack of multisector partnerships (i.e. health services, transport, education, sport/recreation, urban planning sectors) and the absence of clarity about the most effective/feasible strategies] [55]. Furthermore, patients with CVD and/or multiple CVD risk factors require medically supervised programs for exercise training initiations and motivational strategies for long-term adherence [13]. Therefore, although Australia and Brazil provide universal access to a range of community and emergency health services (including hospital care and prescriptions), Australia has one of the most efficient articulated health system worldwide, largely focused on health promotion and disease prevention, rather than on an acute care model [27–30]. These differences could explain why “walking” was 65% more frequent in South Australia than in the Brazilian study, and the fact that individuals at risk or with CVD practicing MVPA were twice more frequent in the NWAHS than in the EpiFloripa study.

This study has some important strengths, including the investigation of two large population-based samples of adults living in two cities from two countries with a similar profile of NCD conditions but different socioeconomic contexts, the use of validated instruments to assess QoL, and standardized methods for data collection and analysis. Nonetheless, some limitations must be recognized. Firstly, the cross-sectional design of the analyses cannot be used to infer causal associations (i.e. being active leading to a higher QoL, or vice versa, or even mutual influence). However, longitudinal results from observational and intervention studies have identified similar findings [12, 13, 21, 53]. Secondly, CVD and most CVD risk factors were identified by self-reported diagnosis. Although we combined that information with the use of medication for these conditions to improve accuracy, these kinds of data have intermediate levels of sensitivity (ranging from 33% to 85%) [56]. Even so, it is unlikely that this source of bias could explain our results, as it would have reduced the magnitude of the associations. Furthermore, although we did not investigate the severity of CVD conditions, some studies have found that patients with mild or severe CVD have a similar impact on QoL [45, 57]. Thirdly, different generic questionnaires were used to measure QoL in each study (WHOQOL-Bref and SF-36) which have a different background, structure, domains, and scales [19, 37]. Despite this limitation, results for the physical domain were consistent in the two studies regarding magnitude and direction of the associations. Finally, PA was assessed considering different instruments in each study, and none of them allowed the investigation of the time/frequency of other important sources of PA (at work, domestic chores, or other forms of low-intensity PA). Nonetheless, these instruments have shown good reliability and validity compared to gold standards in the assessment of leisure PA levels [39–41].

In conclusion, both in Southern Brazil and South Australia, adults at risk or with CVD reported lower QoL compared to healthy individuals, and this deleterious relationship was stronger with the physical compared to the psychological domain. “Walking” time was a more important moderator than other MVPA to mitigate the adverse effects of CVD and their risk factors on QoL, and these results were more consistent in South Australia than in Southern Brazil. Our findings highlight the need to reconsider the recommendations about the forms and intensity of PA indicated for the management and prevention of CVD, as “walking” is also more prevalent in the community and requires less professional advice to be given compared to MVPA among individuals at risk or with CVD. These factors should be considered in the design and implementation of public health interventions aiming to promote PA, improve QoL and reduce the impact of CVD.

## Supporting information

S1 DatasetEpiFloripa Dataset of the EpiFloripa study (Brazil). (XLS)

S1 Fig Flowchart of the EpiFloripa study and North Adelaide Health Study and variables used from each study. The baseline of the EpiFloripa study included participants aged 20–59 years and the baseline of the NWAHS included participants aged 18+ years.
(TIF)

S1 Table Sample characteristics in the baseline and last follow-up of the EpiFloripa study (2012–2013) and NWAHS (2008–2010). (PDF)
