# Supplementary figures and images for: Physical activity moderates the deleterious relationship between cardiovascular disease, or its risk factors, and quality of life: Findings from two population-based cohort studies in Southern Brazil and South Australia
Source: PLoS One. 2018 Jun 7;13(6):e0198769. doi: 10.1371/journal.pone.0198769 (PMC5991645; doi:10.1371/journal.pone.0198769)

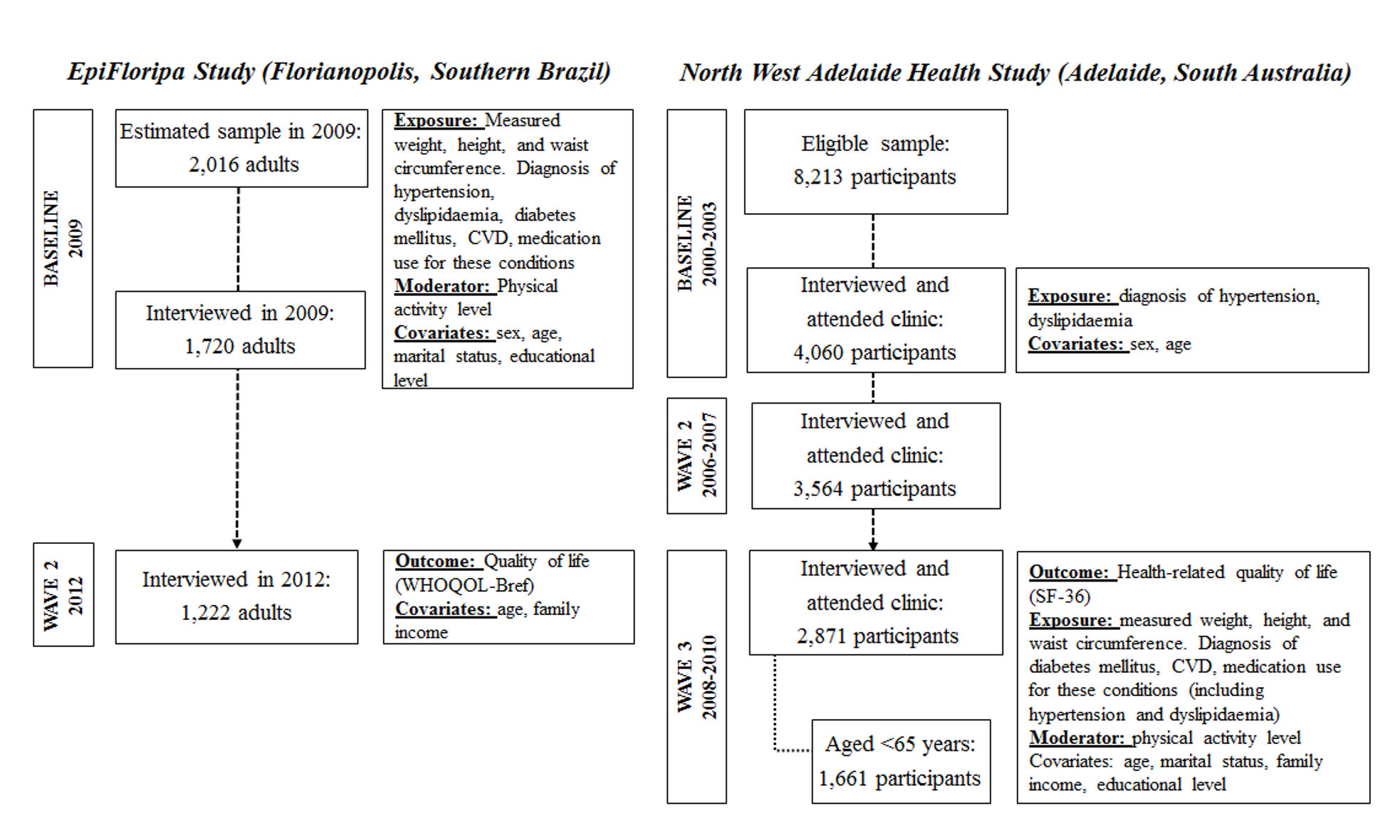

Supplement: S1 Fig — The baseline of the EpiFloripa study included participants aged 20–59 years and the baseline of the NWAHS included participants aged 18+ years. (TIF) [file pone.0198769.s002.tif]
