# Supplementary material for: Physical activity moderates the deleterious relationship between cardiovascular disease, or its risk factors, and quality of life: Findings from two population-based cohort studies in Southern Brazil and South Australia
Source: PLoS One. 2018 Jun 7;13(6):e0198769. doi: 10.1371/journal.pone.0198769 (PMC5991645; doi:10.1371/journal.pone.0198769)
Supplement: S1 Table — (PDF) [file pone.0198769.s003.pdf]

Supplementary Table S1. Comparison of the sample characteristics between the baseline and the last follow-up in the EpiFloripa study (2012-2013) and NWAHS (2008-2009).

	**EPIFLORIPA**		**NWAHS**	
**Baseline characteristics**	**Included in 2009 (N=1,720)**	**Evaluated in 2012* (n=1,222)**	**Included in 2000-03 (N=4,060)**	**Evaluated in 2008-10* (n=2,871)**
Gender (males) - %	48.4	48.2	49.1	48.4
Age (years) - Mean±SD	37.1±11.3	36.0±12.0	44.9±18.2	42.8±16.7
Marital status (married or living with a partner) - %	60.4	57.1	62.2	64.1
Educational level (up to secondary) - %	55.8	55.4	43.1	41.6
Obesity†	15.7	15.5	27.0	25.7

SD = standard deviation

* Comparison considering baseline characteristics of each sample and using the respective sampling weights

† Obesity = body mass index ≥ 30.0 kg/m^2
